# Prevalence and Risk Factors of Metabolic Syndrome among the Homeless in Taipei City: A Cross-Sectional Study

**DOI:** 10.3390/ijerph18041716

**Published:** 2021-02-10

**Authors:** Ming Gu, Chi-Jie Lu, Tian-Shyug Lee, Mingchih Chen, Chih-Kuang Liu, Ching-Lin Chen

**Affiliations:** 1 Graduate Institute of Business Administration, Fu Jen Catholic University, New Taipei City 242304, Taiwan; gm19850617@gmail.com (M.G.); 059099@mail.fju.edu.tw (C.-J.L.); 036665@mail.fju.edu.tw (T.-S.L.); 081438@mail.fju.edu.tw (M.C.); Charles.jhs@gmail.com (C.-K.L.); 2 Artificial Intelligence Development Center, Fu Jen Catholic University, New Taipei City 242304, Taiwan; 3 Department of Information Management, Fu Jen Catholic University, New Taipei City 242304, Taiwan; 4 Department of Urology, Fu-Jen Catholic University Hospital, New Taipei City 242304, Taiwan; 5 Taipei City Hospital, Taipei City 103012, Taiwan

**Keywords:** homeless, metabolic syndrome, dyslipidemia, Taipei, Taiwan

## Abstract

The safety and health of homeless people are important social issues. Metabolic syndrome (MetS) is a sub-health-risk phenomenon that has been severely aggravated worldwide in recent years. The purpose of this study was to investigate the prevalence and risk factors of MetS among the homeless in Taipei City, Taiwan. In this study, a convenience sampling was conducted at homeless counseling agencies in Taipei City from April 2018 to September 2018. A total of 297 homeless participants were recruited, from whom clinical indicators and questionnaire information were collected. Through statistical verification, analysis of variance (ANOVA), and logistic regression, we found the following main conclusions for homeless adults in Taipei: (1) The prevalence of MetS was estimated to be 53%, with 50% meeting four or more diagnostic conditions. (2) Dyslipidemia (high-density lipoprotein (HDL) deficiency and elevated triglyceride (TG)) showed the strongest association with the prevalence of MetS; more than 83% of people with HDL deficiency or hypertriglyceridemia had MetS. For the patient groups meeting more MetS diagnostic conditions, the values of high-density lipoprotein cholesterol (HDL-C), TG, and total cholesterol (TC) increased significantly. (3) The deterioration of MetS was significantly related to the high prevalence of hyperlipidemia (HL). (4) The homeless who were divorced, separated or widowed were more likely to suffer from MetS.

## 1. Introduction

Based on national reports as of the end of 2019, it was estimated that no less than 150 million people in the world, or about two percent of the world’s population, were homeless [1]. Statistics for the same period showed that there were approximately 567,715 homeless people living in the United States [2], and the homeless population in Europe was at least 700,000 [3]. These reports represented the states of homelessness before the COVID-19 crisis began. Unemployment, poverty, and food crisis caused by the pandemic in 2020 have led to the increase in the homeless population worldwide [4,5,6]. This trend may continue and cause the health care of the homeless population to become a huge public health and social problem. The health problems of the homeless mainly originated from poverty and lack of safety, malnutrition, poor hygiene habits, exposure to low temperatures, smoking and alcohol abuse, and lack of medical resources [7,8]. Therefore, they were more likely to suffer from mental illness, respiratory diseases, metabolic syndrome (MetS), cardiovascular disease (CVD), and various infectious diseases, among which MetS was more common in the homeless and was an indirect risk factor of other diseases [9,10].

Metabolic syndrome (MetS) is not a specific disease but a group of risk factors related to metabolism and cardiovascular problems, which are summarized as central obesity, dyslipidemia, elevated blood pressure (BP) and insulin resistance [11,12]. The aggregation of these risk factors would increase the risk of chronic diseases such as coronary heart disease, stroke, renal vascular disease, type 2 diabetes, and the risk of death from diseases [13,14]. Patients with MetS had a greatly increased chance of suffering from cardiovascular disease, and the mortality rate was 1.5 to 2.5 times that of people without MetS [15,16,17,18,19]. From the medical perspective, the determination of MetS can warn patients to prevent many chronic diseases as soon as possible. In recent years, people’s living habits tended to be lack of physical activity, poor eating habits, obesity and other forms, leading to the widespread occurrence of MetS [20,21]. The MetS population also had lower immunity to infectious diseases [22]. It was estimated that the prevalence of MetS in the US adults had ranged from 22% to 34% [23,24]. In most countries in the Asia-Pacific region, nearly one-fifth of adults were affected by MetS, and the prevalence was increasing year by year [25]. According to the latest MetS survey conducted in Taiwan in 2011, between 1993 and 2008, the prevalence of MetS among men in Taiwan rose from 13.6% to 25.5%, and the prevalence among women rose from 26.4% to 31.5% [26].

Homeless people were more susceptible to the threat of metabolic syndrome (MetS), induced chronic diseases and higher mortality [10,27]. However, because it was difficult to convene homeless people for a health survey, there were relatively few studies on MetS of the homeless. Rivas-Vazquez et al. (2011) surveyed 122 homeless adults in South Florida and found that the prevalence of MetS was 29.5%. Elevated waist circumference (WC) in 48.5% and elevated blood pressure (BP) in 44.3% of the population were the two most frequent risk factors for the syndrome [28]. Scott et al. (2013) examined 252 homeless adults in Ireland and found that 22% were patients with MetS, of which the smoking rate was 78%, the drinking rate was 93%, 90% had abdominal obesity, and the patients with MetS were significantly older than those without MetS [29]. A health survey of 257 homeless men in Sydney found that 44% had MetS, 69% were smokers, 62% had a history of chronic alcohol abuse, and 29% had hepatitis C [30]. Baggett et al. (2018) pointed out that depression, anxiety, and a high smoking rate of 68%–80% were the main risk factors for MetS of the homeless [31].

As of May 2020, statistics from the Ministry of Health and Welfare (MOHW) showed there were approximately 3277 homeless people living in Taiwan [32]. Although the homeless population in Taiwan is relatively small, the government and medical circles have always been committed to the development of a welfare society for all. Health care and life counseling for the homeless are important tasks of the MOHW and local social bureaus [33]. We explored the prevalence and risk factors of MetS for homeless people in Taipei City.

## 2. Materials and Methods

### 2.1. Study Design

The research team from Taipei City Hospital conducted a health investigation for homeless adults in Taipei city, in cooperation with Taipei City Social Bureau and homeless counseling agencies in Taipei. The investigation period was from 1 April 2018 to 30 September 2018. Among the homeless accommodated by the homeless counseling agencies, a convenience sampling was conducted, and a total of 297 homeless adults volunteered to participate in the survey, accounting for 44.4% of Taipei city’s homeless population, with sample male-to-female ratio 77.4%:22.6%. According to statistics from the Ministry of Health and Welfare (MOHW) in 2018, the number of homeless in Taiwan was 2603, of which the male-to-female ratio was 89.1%:10.9%; and that in Taipei City was 669 with male-to-female ratio 85.3%:14.7% [32]. The basic distribution of the sample population in this investigation was similar to that of the actual street sample and was sufficiently representative. Informed consent was obtained from each participant after a full explanation of the investigation. This research has been reviewed and approved by the Research Ethics Committee of Taipei City Hospital (Case No: TCHIRB-10805021-E).

#### 2.1.1. Testing Procedures

The following testing procedures were performed in accordance with the relevant body measurement, blood test, and infectious disease recognition standards set by the Ministry of Health and Welfare (MOHW) [34,35,36]. The team of nurses from Zhong-Xing Branch of Taipei City Hospital assisted the homeless adults to complete the tests.

A standardized structured questionnaire was applied for the face-to-face interviews prior to the tests. Participants’ demographic characteristics (gender, age, marital status, and education level) and lifestyle (smoking status, drinking status, betel nut chewing status, and physical activity habits) information was collected. Marital status was divided into never married, married or living together, and divorced/separated/widowed. Educational level was divided into elementary school or lower, junior or senior school, and college or higher. Smoking, drinking and betel nut chewing status were listed as never, former, or current user. Physical activity habits were recorded as days of physical activity per week.

Investigated anthropometric indicators included height, body weight, waist circumference (WC), and hip circumference (HC). Participants were required to remove their shoes and heavy clothes before taking measurements. Height and body weight were recorded to the nearest 0.1 cm and 0.1 kg with an electronic height–weight scale. WC was measured with a flexible steel tape measure placed midway between the lowest rib and iliac crest to the nearest 0.1 cm. HC was measured to the nearest 0.1 cm at the widest part of the hip region.

The participants’ blood indicators were checked through clinical examinations. Blood indicators included systolic blood pressure (SBP), diastolic blood pressure (DBP), fasting plasma glucose (FPG), total cholesterol (TC), triglycerides (TG), high-density lipoprotein cholesterol (HDL-C), hepatitis A (HAV), hepatitis B (HBV), hepatitis C (HCV), syphilis, and human immunodeficiency virus (HIV). Mercury sphygmomanometer was used to measure SBP (mmHg) and DBP (mmHg). The interval between each measurement was 10–15 minutes. Heart rate was measured by pulse, and the unit was beat per minute (bpm). Blood sampling were collected after fasting for at least 8 h. Then, FPG (mg/dl), TC (mg/dl), TG (mg/dl), HDL-C (mg/dl), HAV antibodies, HBV antibodies, HCV antibodies, treponema pallidum antibody, and HIV RNA indicators were checked.

#### 2.1.2. Diagnostic Criteria

According to the diagnostic criteria defined by the Ministry of Health and Welfare (MOHW) [37], the cutoffs for the abnormalities of various indicators that define metabolic syndrome (MetS) were summarized as follows:

I. Central obesity: waist circumference (WC) ≥ 90 cm (male); ≥ 80 cm (female).
II. Hypertriglyceridemia: fasting plasma triglyceride (TG) concentration ≥ 150 mg/dl.
III. High-density lipoprotein (HDL) deficiency: high-density lipoprotein cholesterol (HDL-C) concentration < 40 mg/dl (male); <50 mg/dl (female).
IV. Elevated blood pressure (BP): systolic blood pressure (SBP) ≥ 130 mmHg or diastolic blood pressure (DBP) ≥ 85 mmHg.
V. Insulin resistance: fasting plasma glucose (FPG) concentration ≥ 100 mg/dl.

Patients with 3 or more of the above abnormal indicators can be considered as suffering from MetS.

The cardiovascular disease indicators hypertension (HT), hyperglycemia (HG), and hyperlipidemia (HL) related to MetS were defined according to the cardiovascular diagnostic criteria of the MOHW [38] as follows:

HT: SBP ≥ 140 mmHg or DBP ≥ 90 mmHg.
HG: FPG concentration ≥ 126 mg/dl.
HL: Total cholesterol (TC) concentration ≥ 240 mg/dl or TG ≥ 200 mg/dl.

The determination of HT, HG, HL, and the two MetS indicators of elevated BP and insulin resistance included the use of medicines (except for Chinese herbal medicine) for lowering BP, FPG, or blood cholesterol according to the doctor’s prescription to result in normal test values.

### 2.2. Statistical Analyses

Data were analyzed using R software 3.6.3 (R core team, Vienna, Austria). Descriptive statistics and analyses of differences (Mann-Whitney U test, chi-square test, and Fisher’s exact test) were computed for the clinical indicators, demographic and lifestyle variables, and the prevalence of various clinical diseases including metabolic syndrome (MetS), as well as the differences of these between the sexes in the homeless. Adjusted odds ratio (OR) with 95% confidence interval (CI) was calculated using logistic regression to estimate the association of risk factors with MetS. Welch’s test for analysis of variance (Welch’s ANOVA) was used to determine the differences of cardiovascular indicators among MetS groups of varying severity, with Games-Howell post-hoc tests. Result values were expressed as means and standard deviations, or frequency percentages. Statistical results were significant at *p*-Value < 0.05.

## 3. Results

After data processing, we obtained 275 valid sample data individuals from 297 participants for analysis. The clinical indicators and the prevalence of diseases among the homeless, as well as a comparison between the sexes, are summarized in Table 1. It can be seen that among the 275 homeless participants, 147 persons (53%) suffered from metabolic syndrome (MetS); 74 of them were patients who met four or more diagnostic conditions (DCs), accounting for half of the MetS patients; 29 were severe patients who met all five DCs, accounting for 20% of the patients with MetS. Among all participants, 133 persons (48%) suffered from hypertension (HT), 85 (31%) suffered from hyperglycemia (HG), and 91 (33%) suffered from hyperlipidemia (HL). Meanwhile, there was 1 person with hepatitis A (HAV), 35 persons with hepatitis B (HBV), 31 with hepatitis C (HCV), 7 with syphilis, and 1 person tested positive for human immunodeficiency virus (HIV). The prevalence of these diseases was not significantly different between the sexes among the homeless. For the results of relevant clinical indicators, only high-density lipoprotein cholesterol (HDL-C) and total cholesterol (TC) had significant differences between the sexes. Statistics of demographic variables shows that 43% of the homeless participants had an education level of elementary school or below, and female homeless had even lower levels of education. 47% of the homeless were unmarried, and 45% were divorced, separated, or widowed. The unmarried ratio of homeless men was significantly higher than that of homeless women. Statistics on lifestyles shows that the smoking rate, drinking rate, and betel nut chewing rate of the homeless were 56%, 24%, and 12%, respectively.

Table 2 shows the pathogenicity distributions of metabolic syndrome (MetS) indicators in the homeless. Among the five diagnostic criteria of MetS, the homeless with high-density lipoprotein (HDL) deficiency or hypertriglyceridemia had a higher prevalence of MetS, at 84% and 83% respectively. Even in people with normal HDL-C or TG indicator, the HDL-C and TG values of MetS patients were also significantly different from those in non-metabolic syndrome (non-MetS) groups. It can be seen that among the MetS indicators, the decrease of HDL-C, the increase of TG, or HDL deficiency and hypertriglyceridemia, had more significant association with the prevalence of MetS.

Table 3 presents the results of the logistic regression for metabolic syndrome (MetS). After adjusting for gender, age, education level, marital status, smoking, drinking, betel nuts chewing status, and physical activity, the adjusted odds ratios (ORs) and 95% confidence intervals (CIs) for MetS for each standard deviation (SD) increase in TC and heart rate were calculated as 1.70 (1.30–2.28) and 1.32 (1.03–1.70). For analyses of multi-category variables of demography and lifestyle, the lowest (commonly worst) level of performance was fixed as a reference in the regression (OR = 1.00). The results show that compared with the unmarried group, the adjusted ORs and 95% CIs of the divorced/separated/widowed group for MetS were 1.72 (1.02–2.93).

Table 4 shows that among the homeless population, 63% of metabolic syndrome (MetS) patients had HT, 42% of MetS patients had HG, and 53% of MetS patients had HL. The results of Welch’s ANOVA showed that the values of TG and TG of MetS patients who met four or five diagnostic conditions (DCs) were significantly higher than those who met three DCs, while the HDL-C values of MetS patients with more DCs were significantly lower than those with less DCs. The results of chi-square analysis showed that there was a significant association between more severe MetS and high prevalence of HL. The numerical trends show that the population with severe MetS also had higher prevalence of HT and HG, but these had not passed the statistical test.

## 4. Discussion

The current study was the first cross-sectional data research on metabolic syndrome (MetS) in the homeless population in Taiwan. Based on results, we found that the prevalence of MetS among the homeless in Taipei was 53%, those meeting four or more diagnostic conditions (DCs) accounted for 50% of the MetS patients, while patients meeting all five DCs accounted for 20%. Compared with the results of previous studies, the prevalence of MetS among the homeless in South Florida of US, Ireland, and Sydney of Australia was 29.5%, 22% and 44%, respectively [28,29,30]. Moreover, among the homeless patients with MetS in South Florida, the proportion meeting four DCs was only 7%, while the number of sample individuals meeting all five DCs was 0 [28].

To further explore the prevalence characteristics of MetS among the homeless in Taipei City, we found that high-density lipoprotein cholesterol (HDL-C) and triglycerides (TG) indicators were more sensitive to the diagnosis of MetS than other three indicators (waist circumference (WC), blood pressure (BP), fasting plasma glucose (FPG)). High-density lipoprotein (HDL) deficiency and elevated TG had more significant association with the prevalence of MetS. On the other hand, for patients with MetS who met more DCs, the values of HDL-C, TG, total cholesterol (TC), and the prevalence of hyperlipidemia (HL) had increased significantly. However, the increases in values of SBP, DBP, FPG, the prevalence of hypertension (HT) and hyperglycemia (HG) had no significant association with MetS levels. Previous studies usually proved that there was the strongest association between MetS and diabetes [17,18,20,21]. Matsuzawa (2005) found that among the five diagnostic indicators of MetS for Japanese, abdominal obesity had the highest prevalence compared with the other four indicators [39]. Kuk and Ardern (2010) pointed out that avoiding HDL deficiency and elevated FPG could more effectively avoid MetS [40]. Devers et al. (2016) and Mohammadifard et al. (2017) indicated that monitoring WC, BP, and FPG was an effective process to prevent MetS [41,42]. Chen and Chen (2018) found that for Taiwanese, maintaining TG values for middle-aged and elderly people and WC values for young people at low levels was the most effective way to prevent MetS [43]. This study found that for the homeless in Taipei City, MetS showed significantly association with the risk factors of dyslipidemia (HDL deficiency, elevated TG, and elevated TC) and the prevalence of HL.

The homeless adults who were divorced, separated or widowed were more likely to suffer from MetS. In Taipei City, 47% of the homeless were unmarried, and 45% were divorced, separated, or widowed. The smoking rate among the homeless adults in Taipei was 56%, and the drinking rate was only 24%. Among the Sydney homeless adults, 69% smoked and 62% had a history of chronic alcohol abuse [30]. The smoking rate of Ireland homeless adults was 78%, and the drinking rate was 93% [29]. Relatively speaking, the smoking and drinking rates of Taipei homeless adults were lower. The current results did not show any significant association between lifestyle variables and MetS. The sample size was limited by the discrete distribution of the homeless, and it was difficult to convene more participants to take part in the survey. There are other limitations in this study. We used a cross-sectional study design, which was unable to prove causality. Moreover, we determined MetS, HT, HG and HL according to the diagnostic criteria, without asking participants whether they had received medication control or treatment, so that the prevalence might be slightly underestimated.

Statistics on the causes of death in Taiwan in 2019 showed that heart disease, cerebrovascular diseases, diabetes and HT accounted for four of the top ten causes of death [44], and these diseases all might be related to MetS. It can be speculated that the risk of MetS for the homeless in Taiwan should be more serious than that of ordinary people. This means that the high prevalence of MetS among the homeless adults in Taipei might cause further health crises, which should arouse the attention of the government and medical departments.

## 5. Conclusions

Among the homeless adults in Taipei, Taiwan, the prevalence of metabolic syndrome (MetS) was estimated to be 53%, which was higher than the statistical results in other parts of the world. Dyslipidemia risks (high-density lipoprotein (HDL) deficiency, elevated triglycerides (TG), and elevated total cholesterol (TC)) were the most important risk factors for MetS. Moreover, the deterioration of MetS was significantly related to the high prevalence of hyperlipidemia (HL). Homeless adults who were divorced, separated or widowed were more likely to suffer from MetS. We hope that the results would provide clinical references and cause the government and medical institutions to pay more attention to the health risks of the homeless population.

## Figures and Tables

**Table 1:** Statistical description and analysis of difference under gender grouping.

Variables	Total	Men	Women	*p*-Value
No. of subjects	275	213	62	
WC (cm)	90.0 ± 12.7	90.1 ± 12.8	89.4 ± 12.3	0.75 ^a^
SBP (mmHg)	138.0 ± 22.9	137.3 ± 22.9	140.4 ± 22.9	0.27 ^a^
DBP (mmHg)	84.2 ± 14.5	84.5 ± 14.3	83.2 ± 15.2	0.35 ^a^
FPG (mg/dl)	135.5 ± 89.3	138.4 ± 90.9	125.7 ± 83.6	0.17 ^a^
TG (mg/dl)	196.7 ± 177.9	197.3 ± 180.3	194.7 ± 170.8	0.98 ^a^
HDL-C (mg/dl)	46.2 ± 13.3	44.3 ± 12.3	52.6 ± 14.9	<0.001 ^a^
TC (mg/dl)	172.9 ± 37.4	168.3 ± 34.6	188.8 ± 42.0	<0.001 ^a^
Heart rate (bpm)	84.7 ± 13.7	85.2 ± 13.8	82.8 ± 13.3	0.37 ^a^
MetS	147 (53%)	110 (51%)	37 (60%)	0.33 ^b^
five DCs	29 (10%)	20 (9%)	9 (15%)	
four DCs	45 (16%)	32 (15%)	13 (21%)	
three DCs	73 (27%)	58 (27%)	15 (24%)	
HT	133 (48%)	102 (48%)	31 (50%)	0.88 ^b^
HG	85 (31%)	71 (33%)	14 (23%)	0.15 ^b^
HL	91 (33%)	65 (31%)	26 (42%)	0.13 ^b^
HAV	1 (0.4%)	0 (0%)	1 (1.6%)	0.23 ^c^
HBV	35 (13%)	30 (14%)	5 (8%)	0.3 ^b^
HCV	31 (11%)	25 (12%)	6 (10%)	0.82 ^b^
Syphilis	7 (2.5%)	5 (2.3%)	2 (3%)	0.66 ^c^
HIV	1 (0.4%)	1 (0.5%)	0 (0%)	1 ^c^
Age	59.74 ± 12.23	59.27 ± 11.86	61.37 ± 13.40	0.38 ^a^
Education level				0.04 ^b^
Elementary school or lower	118 (43%)	83 (39%)	35 (56%)	
Junior or senior school	134 (49%)	112 (53%)	22 (36%)	
College or higher	23 (8%)	18 (8%)	5 (8%)	
Marital status				0.005 ^b^
Never married	128 (47%)	108 (51%)	20 (32%)	
Married or living together	22 (8%)	12 (5%)	10 (16%)	
Divorced/separated/widowed	125 (45%)	93 (44%)	32 (52%)	
Smoking status				<0.001 ^c^
Never	102 (37%)	56 (26%)	46 (74%)	
Former	20 (7%)	19 (9%)	1 (2%)	
Current	153 (56%)	138 (65%)	15 (24%)	
Drinking status				<0.001 ^c^
Never	170 (62%)	113 (53%)	57 (92%)	
Former	38 (14%)	37 (17%)	1 (2%)	
Current	67 (24%)	63 (30%)	4 (6%)	
Betel nuts chewing				<0.001 ^c^
Never	204 (74%)	147 (69%)	57 (92%)	
Former	38 (14%)	38 (18%)	0 (0%)	
Current	33 (12%)	28 (13%)	5 (8%)	
Physical activity	3.53 ± 3.09	3.54 ± 3.07	3.50 ± 3.18	0.80 ^a^

WC: waist circumference; SBP: systolic blood pressure; DBP: diastolic blood pressure; FPG: fasting plasma glucose; TG: triglycerides; HDL-C: high-density lipoprotein cholesterol; TC: total cholesterol; MetS: metabolic syndrome; DC: diagnostic condition; HT: hypertension; HG: hyperglycemia; HL: hyperlipidemia; HAV: hepatitis A; HBV: hepatitis B; HCV: hepatitis C; HIV: human immunodeficiency virus. ^a^ The P-value represents the statistical significance of difference in means and standard deviations. ^b^ The P-value represents the statistical significance of the chi-square test results. ^c^ The P-value represents the statistical significance of the Fisher’s exact test.

**Table 2:** Pathogenicity distributions of metabolic syndrome (MetS) indicators.

MetS Indicators	Total	Non-MetS	MetS	*p*-Value
No. of subjects	275	128	147	
WC				<0.001 ^b^
Normal	126 (46%)	93 (74%)	33 (26%)	
		78.68 ± 6.90	81.79 ± 5.50	0.033 ^a^
Central obesity	149 (54%)	35 (23%)	114 (77%)	
BP				<0.001 ^b^
Normal	97 (35%)	68 (70%)	29 (30%)	
SBP		114.53 ± 9.51	117.90 ± 7.52	0.17 ^a^
DBP		71.94 ± 7.23	73.69 ± 5.56	0.30 ^a^
Elevated BP	178 (65%)	60 (34%)	118 (66%)	
FPG				<0.001 ^b^
Normal	118 (43%)	83 (70%)	35 (30%)	
		86.0 ± 9.62	87.7 ± 8.29	0.46 ^a^
Insulin resistance	157 (57%)	45 (29%)	112 (71%)	
HDL-C				<0.001 ^b^
Normal	165 (60%)	110 (67%)	55 (33%)	
		55.22 ± 11.66	50.85 ± 10.45	0.008 ^a^
HDL deficiency	110 (40%)	18 (16%)	92 (84%)	
TG				<0.001 ^b^
Normal	145 (53%)	106 (73%)	39 (27%)	
		96.45 ± 27.28	111.69 ± 29.02	0.002 ^a^
Hypertriglyceridemia	130 (47%)	22 (17%)	108 (83%)	

WC: waist circumference; SBP: systolic blood pressure; DBP: diastolic blood pressure; FPG: fasting plasma glucose; TG: triglycerides; HDL-C: high-density lipoprotein cholesterol; MetS: metabolic syndrome; Non-MetS: non-metabolic syndrome. ^a^ The P-value represents the statistical significance of difference in means and standard deviations. ^b^ The P-value represents the statistical significance of the chi-square test results.

**Table 3:** Multivariate adjusted odds ratios (ORs) for metabolic syndrome (MetS) after adjustment for potential confounders.

Variables	Unadjusted			Adjusted		
	OR	95%CI	*p*-Value	OR	95%CI	*p*-Value
TC	1.01	1.01–1.02	<0.001	1.01	1.01–1.02	<0.001
Heart rate	1.02	1.00–1.04	0.042	1.02	1.00–1.04	0.031
Age	1.01	0.99–1.03	0.40	1.00	0.98–1.02	0.88
Education level						
Elementary school or lower	1.00	—	—	1.00	—	—
Junior or senior school	1.08	0.65–1.77	0.77	1.25	0.71–2.19	0.44
College or higher	0.67	0.27–1.65	0.39	0.75	0.29–1.90	0.55
Marital status						
Never married	1.00	—	—	1.00	—	—
Married or living together	0.76	0.29–1.89	0.56	0.57	0.20–1.59	0.29
Divorced/separated/widowed	1.76	1.07–2.92	0.03	1.72	1.02–2.93	0.04
Smoking status						
Never	1.00	—	—	1.00	—	—
Former	0.92	0.35–2.44	0.87	0.79	0.28–2.25	0.66
Current	1.13	0.68–1.86	0.65	1.37	0.77–2.46	0.29
Drinking status						
Never	1.00	—	—	1.00	—	—
Former	0.65	0.32–1.32	0.24	0.71	0.33–1.51	0.37
Current	0.94	0.53–1.66	0.83	0.99	0.53–1.83	0.97
Betel nuts chewing status						
Never	1.00	—	—	1.00	—	—
Former	0.72	0.35–1.44	0.35	0.81	0.39–1.67	0.56
Current	1.78	0.84–3.98	0.15	1.92	0.87–4.47	0.12
Physical activity	0.95	0.88–1.02	0.18	0.94	0.88–1.03	0.196

OR: odds ratio; CI: confidence interval; MetS: metabolic syndrome; TC: total cholesterol.

**Table 4:** Comparison of cardiovascular disease (CVD) risks in patients with different degrees of metabolic syndrome (MetS).

Variables	MetS Patients	Three DCs (III)	Four DCs (IV)	Five DCs (V)	*p*-Value	Games-Howell Test
No. of subjects	147	73	45	29		
SBP		145.3 ± 24.91	142.2 ± 18.62	151.8 ± 17.37	0.09 ^a^	——
DBP		87.0 ± 16.02	85.6 ± 12.56	92.5 ± 15.62	0.14 ^a^	——
FPG		137.2 ± 89.83	164.7 ± 108.1	173.9 ± 95.14	0.14 ^a^	——
HDL-C		44.44 ± 11.37	39.11 ± 10.56	34.17 ± 6.08	<0.001 ^a^	III > IV > V
TG		188.6 ± 105.6	317.4 ± 248.3	334.1 ± 202.7	<0.001 ^a^	III < IV, V
TC		167.9 ± 33.25	195.0 ± 49.08	191.5 ± 31.92	<0.001 ^a^	III < IV, V
HT	92 (63%)	43 (59%)	26 (58%)	23 (79%)	0.11 ^b^	
HG	62 (42%)	27 (37%)	18 (40%)	17 (59%)	0.13 ^b^	
HL	78 (53%)	24 (33%)	27 (60%)	27 (93%)	<0.001 ^b^	

DC: diagnostic condition; SBP: systolic blood pressure; DBP: diastolic blood pressure; FPG: fasting plasma glucose; HDL-C: high-density lipoprotein cholesterol; TG: triglycerides; TC: total cholesterol; HT: hypertension; HG: hyperglycemia; HL: hyperlipidemia. ^a^ The P-value represents the statistical significance of difference in means and standard deviations. ^b^ The P-value represents the statistical significance of the chi-square test results.

## Data Availability

The data are not publicly available due to privacy and ethical restrictions.
